# The relationship between family variables and family social problems during the COVID-19 pandemic

**DOI:** 10.1371/journal.pone.0270210

**Published:** 2022-06-29

**Authors:** Saeko Kamoshida, Naoto Nihonmatsu, Gen Takagi, Koubun Wakashima

**Affiliations:** 1 Graduate School of Education, Tohoku University, Sendai, Japan; 2 Research Fellow, Japan Society for the Promotion of Science (JSPS), Tokyo, Japan; 3 Faculty of Comprehensive Welfare, Tohoku Fukushi University, Sendai, Japan; 4 Tohoku University, Sendai, Japan; Satyawati College (Eve.), University of Delhi, INDIA

## Abstract

This study examined the relationship between variables about family members co-residing during the COVID-19 pandemic and anxiety about COVID-19, domestic violence from spouse, child abuse anxiety, internet addiction, and mental health as social problems related to the COVID-19 pandemic. A total of 220 parents (70 male and 150 female, age; *M* = 41.6, *SD* = 34.4) were included in the analysis. Stepwise hierarchical multiple regression analysis was conducted with dependent variables of fear of COVID-19, spousal violence, anxiety regarding perpetrating child abuse, internet addiction, and mental health. The independent variables were basic variables related to family members such as family composition. The results demonstrated that parents with preschool children were anxious about the possibility that they might abuse their children (*β* = .203, *p* < .01). Subjects who smoked were associated with anxiety about being the victim of domestic violence by their spouse (*β* = .154, *p* < .05). Those whose income had decreased due to the COVID-19 pandemic, those who were employed, and those with few rooms in their house were more likely to be dependent on the Internet (in order, *β* = .189, *p* < .01; *β* = .196, *p* < .01; *β* = -.140, *p* < .05). Finally, mental health was impaired among those whose income was reduced by the COVID-19 pandemic (*β* = .134, *p* < .05) and among those who had conflicting opinions in their families regarding the pandemic (*β* = .206, *p* < .01). These results indicate that family variables are associated with family social problems. Additionally, we assume these have been exacerbated by the COVID-19 pandemic. While further research is required to determine the causal relationships among the variables, the findings can be used as an indicator of support that should be provided to families.

## Introduction

The world is in the midst of a pandemic due to the spread of a new coronavirus (hereafter referred to as COVID-19). In addition to medical measures, the Japanese government has limited the flow of people in and out of cities, regions, and in some cases, countries, by locking down cities, introducing remote work systems such that only necessary employees can be physically present in the workplace, and closing schools. As a result, most people have faced some form of restriction of movement for over two years. As this has led people to spend more time with their families, family related problems have increased. Specifically, social trends include increases in child abuse, Internet addiction, problems related to nursing care [1–3], consultations regarding domestic violence and divorce [4], and suicide rates [5].

In Japan, there have not been many psychological studies focusing on social trends and the effects of COVID-19. However, if foreign studies are included, such as restrictions on going out and remote work due [6–8], anxiety and coping [9–11], and discrimination against infected people [12], fluctuation in intra-family communication and its association with preventive behavior [13–15], and children who spend more time looking at screens and less time exercising [16–18]. Several studies have shown that frequent intrafamily communication and parents telling their children about the risk of the virus may promote preventive behaviors within the family [14,15]. In one other study, parents of elementary school-aged children reported increases in irregular sleep, disordered eating habits, and use of games and smartphones among their children during the COVID-19 pandemic, Especially, disordered eating habits were related to stress responses such as psychosomatic symptom, depression and anxiety, anger, lacking energy [16].

Thus, COVID-19 social problems such as domestic violence, anxiety, and Internet addiction have not been examined. In the international literature focusing on these social trends, based on past experiences with SARS-CoV-2, Ebola, hurricanes, and water disasters, Usher et al. [19] argue incidentally that violence against women and children increases during disasters. According to Madanes and Madanes [20], problems such as economic deprivation, sexual problems, domestic violence, and abuse in the family are interrelated as deviations from the rules lead to other deviations, which can lead to a variety of problems. However, while previous studies have illustrated the impact of COVID-19 on families from various aspects, they have not examined the social trends of domestic violence, child abuse, and mental health during the COVID-19 pandemic.

To address the limitations, we need to include not only macro variables such as housing and economic conditions, but also various micro variables within the family, such as number of household members, family composition, time spent together, and number of rooms in the house, and their impact on social issues. Therefore, the purpose of this study was to examine the relationship between variables about family members co-residing during the COVID-19 pandemic and anxiety about COVID-19, domestic violence and child abuse, Internet addiction, and mental health.

## Materials and methods

This study was conducted in October 2021. In Japan, the average weekly number of patients infected with COVID-19 in early October 2021 was 1,810. The infection rate had decreased from the average weekly rate of 23,149 in August 2021, at the time of the fifth wave of the pandemic in Japan. The declaration of the state of emergency was subsequently lifted in October 2021 [21].

Of 234 parents with children (74 males and 160 females, age; *M* = 41.7, *SD* = 34.3), a total of 220 (70 males, 150 females; *M* = 41.6, *SD* = 34.4) were included in the analysis, after excluding two participants whose data were rated as of low quality according to Masuda’s operation check [22], which assesses whether participants read statements correctly.

The first author administered the questionnaire through a web-based survey company that targets for all Japanese people. Potential participants were informed that they were free to answer or not answer the questions as they wished, how personal information would be managed, and that referral to a consultation service was available for psychological problems arising from participation; participants who provided informed consent were recruited into the study.

Variables related to the study participants and their families, such as gender and age, were set as the key variables. These data consisted of sex; age; nationality; prefecture of residence; city, town or village of residence; smoking history; presence of respiratory disease currently being treated; disease other than respiratory disease currently being treated; mental disease currently being treated (anxiety disorder, depression, none, other); name of disease other than respiratory disease and mental disease currently being treated; occupation presence or lack thereof; and increase or decrease in income during the COVID-19 pandemic.

Family information collected included presence or absence of co-residence with family, number of family members, time spent with family members, number of rooms in the home, presence of older adults in the family, presence of pregnant women in the family, presence of preschool children in the family, presence of persons with respiratory diseases in the family, family members who were health care workers, presence of persons infected with COVID-19 including those previously infected, presence of unvaccinated persons, and presence of conflicting opinions about COVID-19 in the family.

Anxiety about COVID-19 was measured using the Fear of COVID-19 Scale—Japanese Version (FCV-19S-J) [9], which contains seven items, each of which is rated five-point scale. Frequency of domestic violence was measured using the Violence Against Women’s Screen [23], which consists of seven items presented in a three-part format, with a cutoff score of nine points denoting serious violence. Each item starts with “Your partner is” so that it can be used regardless of gender. Anxiety about perpetrating child abuse was measured using the Child Abuse Anxiety Scale [24], which comprises of 17 items and four methods regarding abuse anxiety, such as “I feel that I will eventually become very violent toward my children” and “I worry that others will think that I am also abusive.” The Japanese version of the Internet Addiction Test [25], which is based on a translation of Young’s (1996) Internet addiction test (IAT), was used to measure Internet addiction [26]. Mental health status was examined using the K6 [27], a 6-item, 5-point scale.

The first section of the survey stated its purpose. It also stated that participation was voluntary, the survey was anonymous, and personal information would not be disclosed to third parties. Only those who agreed to participate in the survey were able to complete the questionnaire. Additionally, The Tohoku University Graduate School of Education’s Ethics Committee granted ethical approval for this study (ID: 21-1-032).

IBM SPSS Statistics 28 was used for the statistical analysis. Ten basic dummy variables were created to classify participants as female, with a history of smoking, with diseases other than respiratory disease, with preschool children, a health care worker, infected or previously infected with coronavirus, employed, having lost income during the pandemic period, being vaccinated against COVID-19, and with presence of family conflict. The six variables with extremely skewed frequencies were excluded from the analysis, namely presence of respiratory disease, presence of mental illness, presence of older adult, presence of pregnant woman, presence of person, infected or previously infected with COVID-19, and presence of family member with respiratory disease. Composite scores were used in accordance with previous studies for the FCV-19S [9], the Violence Against Women Screen [23], the Child Abuse Anxiety Scale [24], and the Japanese version of the Internet Addiction Test [25], and the K6 [27]. Hierarchical stepwise multiple regression analysis was then conducted with each of five composite scores as the dependent variables in separate analyses with and the independent variables consisting of the basic variables. In Step 2, seven basic variables related to family members were introduced sequentially: number of people living together, shared time, number of rooms, presence or absence of preschool children, presence or absence of medical personnel, presence or absence of vaccinees, and presence or absence of family conflicts related to COVID-19.

## Results

The frequency distribution of the basic variables pertaining to the participants in this study is presented in Table 1. Less than 10% of participants reported a positive response for six variables (presence of respiratory diseases, presence of psychiatric diseases, presence of older adults, presence of pregnant women, presence of persons currently or previously infected with COVID-19, and presence of family members with respiratory diseases). The descriptive statistics for each scale are illustrated in Table 2.

**Table 1:** Socio-demographic information (*N* = 220).

Variables	*N* = 220	%	Variables	*N* = 220	%
Gender			Spending time with family		
Male	70	32%	One day	66	30%
Female	150	68%	Half day	47	21%
Age			8 hours	67	30%
10–19	0	0%	6 hours	40	18%
20–29	25	11%	Number of rooms		
30–39	92	42%	1–2	19	9%
40–49	69	31%	3	80	36%
50–59	32	15%	4	62	28%
60-	2	1%	5	44	20%
Smoking status			>6	15	7%
Yes	54	25%	Presence of elderly people		
No	166	75%	Yes	14	6%
Presence of respiratory disease			No	206	94%
Yes	6	3%	Presence of pregnant woman		
No	214	97%	Yes	4	2%
Presence of other diseases			No	216	98%
Yes	27	12%	Presence of preschoolers		
No	193	88%	Yes	100	45%
Presence of mental illness			No	120	55%
Yes	13	6%	Presence of medical personnel		
No	207	94%	Yes	24	11%
Employed			No	196	89%
Yes	150	68%	Presence of COVID-19 infected persons		
No	70	32%	Yes	5	2%
Increase/decrease in income			No	215	98%
Increased or same	160	73%	Presence of unvaccinated persons		
Decreased	60	27%	Yes	51	23%
Number of cohabitants			No	169	77%
2	14	6%	Conflicting opinions about COVID-19 in the family		
3	115	52%	Yes	42	19%
4	65	30%	No	178	81%
>5	26	12%	Family members with respiratory diseases		
			Yes	14	6%
			No	206	94%

**Table 2:** Descriptive statistics.

Variables	*Minimum*	*Maximum*	*Mean*	*Standard* *Deviation*
Fear of COVID-19 Scale Japanese version (FCV-19S-J)	7	30	17.4	4.98
Violence Against Women Screening Scale	7	19	8.9	2.37
Child Abuse Anxiety Scale	17	68	24.3	9.28
Internet addiction	20	79	37.4	13.53
Mental health (K6)	0	24	5.0	5.17

A hierarchical multiple regression analysis did not identify a significantly predictive model for the FCV-19S J scores. However, the analysis of the violence against women screening scores led to significant *R*^2^ value with a significant smoking (*R*^*2*^ = .019, *β* = .154,95CI = 0.12–1.57, *p* < .05). That is, being a smoker was associated with the perceived risk of being subjected to domestic violence. Among the 54 participants who reported smoking, 28 were male and 26 were female. Having a preschooler was also associated with anxiety that parents might abuse their children (*R*^*2*^ = .037, *β* = .203, 95CI = 1.34–6.20, *p* < .01).

For the Internet addiction test (Table 3), the variance explained for step 3 was the highest; the R^2^ value was significant, as was its increment from step 2 was also significant (*⊿R*^*2*^ = .019, *p* < .05). Internet addiction was associated with decreased income following the pandemic (*β* = .196, *p* < .01), being employed (*β* = .189, *p* < .01), and living in a home with fewer rooms (*β* = -.140, *p* < .05).

**Table 3:** 

		*β*	95%Cl lower limit	95% Cl upper limit
Step 1	Employed (dummy variable)	.203**	2.11	9.68
	*R* ^*2*^	.037**		
Step 2	Employed (dummy variable)	.188**	1.70	9.21
	Loss of income (dummy variables)	.168*	1.17	9.03
	*R* ^*2*^	.061***		
Step 3	Employed (dummy variable)	.189**	1.76	9.21
	Loss of income (dummy variables)	.196**	1.97	9.93
	Number of rooms	-.140*	-3.39	-0.12
	*R* ^*2*^	.076***		

*p < .05,

**p < .01,

***p < .001.

These results indicate that persons whose income decreased versus before the pandemic and those who were employed were more likely to report Internet addiction, while those with more rooms in their house were less likely to report Internet addiction. Note that most of the unemployed persons were women.

Table 4 shows that the coefficient of determination of step 2 of the model for K6 scores was the highest; the R^2^ value was significant, as was its the increment from step 1 (*ΔR*^2^ = .042, *p* < .01). Larger K6 scores were associated with decreased income following the pandemic (*β* = .134, *p* < .05) and the presence of family conflict regarding COVID-19 (*β* = .206, *p* < .01).

**Table 4:** 

		*β*	95%Cl lower limit	95% Cl upper limit
Step 1	Loss of income (dummy variables)	.142*	0.11	3.17
	*R* ^*2*^	.016*		
Step 2	Loss of income (dummy variables)	.134*	0.04	3.05
	Family conflict (dummy variables)	.206**	1.00	4.40
	*R* ^*2*^	.054***		

*p < .05,

**p < .01,

***p < .001.

## Discussion

This study, we examined the relationship between variables about family members co-residing during the COVID-19 pandemic and anxiety about COVID-19, domestic violenceanxiety regarding perpetrating child abuse, Internet addiction, and mental health. First, Individual and family variables such as occupation and the family member living together did not affect anxiety regarding COVID-19. This may be because that the public has become accustomed to the lifestyle caused by the pandemic. A longitudinal study of infection anxiety [28] demonstrated that infection anxiety was highest in December 2020, when the number of newly infected people increased rapidly as part of the third wave in Japan, but decreased in March 2021, when the number of newly infected people decreased. In response to these results, it has been suggested that the weakening of infection anxiety may be due to habituation to the COVID-19 pandemic, such as habituation to infection prevention measures, along with a decrease in crisis awareness after the period of high infection spread. Therefore, it is possible that no difference in infection anxiety was not significantly associated with any study variables because the survey was conducted during a period of decreased infections in Japan.

Being a smoker was associated with the perceived risk of being subjected to domestic violence. Smoking has been associated with severe respiratory failure due to COVID 19 [29]. Many smokers are aware of the risk [30]. Furthermore, smoking has been criticized by the public because it often occurs in crowded situations, in enclosed spaces, with unmasked persons [31]. Thus the public may be more nervous about smoking than before the spread of COVID-19 for three reason: first, smokers are more likely to be in close contact with others when using smoking areas; second, they are more likely than non-smokers to bring COVID-19 into the home; and third, passive smoking increases the risk of serious illness if a family member is infected with COVID-19. That is, smoking can cause marital conflict, which may lead to verbal abuse and violence in some cases. The location of the respondents’ smoking areas was also not disclosed; thus, it is unclear whether smoking is actually a trigger for family conflicts.

Having a preschooler was also associated with anxiety that parents might abuse their children. The Ministry of Health, Labor and Welfare of Japan has indicated summarized that 43.5% of abused children are under six years old [32,33]. Mothers with children between newborn and preschool age were also the highest percentage of respondents (62.3%) who stated that discipline was a factor in child-rearing anxiety. A survey of mothers of infants and toddlers [34] demonstrated that there are conflicts in child rearing, such as regarding what constitutes good and bad discipline. We speculate that these conflicts may become more pronounced during the preschool years when child rearing is difficult; for this reason, the rate of abuse directed toward preschoolers is relatively high. It is highly likely that this is a general tendency, not simply an effect of the COVID-19 pandemic.

It was suggested that Internet addiction may occur among those who are currently working, those who have a lost income due to the COVID-19 pandemic, and those with fewer rooms in their house. First, it has been indicated that there is a relationship between Internet addiction and the presence or absence of interference with social life [35–37]. However, the opposite results were found in the current study, as being employed was associated with higher Internet addiction scores. This may be because the social life of due to some people is not hindered by unemployment (e.g., homemakers and students). For example, during the COVID-19 pandemic, being unemployed may reduce the risk of Internet addiction interfering with social life because issues with interpersonal relationships and activity may be alleviated by virtual contact (e.g., video chat).

It was also found that reduced income was associated with increased Internet addiction. However, it is necessary to consider indirect effects when interpreting the relationship between economic variables, such as income, and psychological variables. A survey by the Ministry of Internal Affairs and Communications of Japan reported that the percentage of Internet users in households with an annual household income of 4 million yen or more exceeded 80% [38]. That is, a possible mechanism is that wealthier households simply own more smartphones and computers and have greater Internet access, which leads their addiction scores to appear higher. In contrast, during the COVID-19 pandemic, adolescents of lower socioeconomic status are at higher risk of problematic Internet use than are those of wealthier socioeconomic status [39]. Taking all of these factors into consideration, loss of income due to reduced work hours following the COVID-19 pandemic is a leading risk factor for Internet addiction or hikikomori.

Finally, it was demonstrated that having fewer rooms at home was associated with increased Internet addiction scores. With respect to this result, rather than considering the relationship between housing type and individual Internet addiction, it is necessary to consider attributes factors such as economic status as a third variable. According to the Ministry of Internal Affairs and Communications of Japan, the percentage of persons who rent accommodations is greatest among in their 20s, and the percentage of persons in owner-occupied houses increases among those aged 30 years older [40]. That is, it is likely, if we assume that the number of rooms at home increases with age, income, and other social status, which is consistent with previous studies [41,42] that illustrate Internet addiction is serious among young people. Additionally, if the number of rooms is large, Internet use was controlled to some extent by their roommates’ lives. However, no association between age and IAT was found in the data of this study. The reason may be that the COVID-19 pandemic has affected the increase in time spent at home and working from home. Therefore, we believe that this is because the rate of Internet use increased equally regardless of age.

Next, it was suggested that mental health may have been impaired among persons whose income had decreased following the COVID-19 pandemic and among whose family members had conflicting opinions regarding COVID-19. Decrease income typically reduces the standard of living and may increase the difficulty of maintaining adequate health. Bosako and Hoshi suggested that the amount of income may mediate the sense of happiness and life satisfaction and contribute to subjective health [43]. Thus, there is a clear relationship between living standards and health based on income. However, as the current data were not panel data, it is possible that the causal relationship may be reversed, such as a decrease in income due to poor mental health. Regarding conflicts among family members, there may be differences in awareness of the need for COVID-19 infection prevention among different age groups and educational levels, as well as differences in awareness of the need for vaccines. For example, study have shown that parents and their children are often divided on the pros and cons of vaccination [44,45]. Furthermore, Yigit et al. [45] reported differences according to gender and educational level. Therefore, we suggest that individual intentions are strongly reflected in vaccine decision-making, especially in the case of COVID-19. This may easily lead to conflicts within families, such as between couples, generations, and between generations of children. Such conflicts of opinion may increase tension within the family and affect the mental health of individuals.

### Limitations

The primary limitation of this study is that it only examined effects at a single point in time; thus, it was not possible to determine causality, namely, whether the effects were caused by the COVID-19 pandemic. Additionally, it is necessary to carefully examine whether socioeconomic and psychological variables are directly related to each other or indirectly related to each other, such as through mediating variables. Further, some items were excluded from the analysis, such as presence of respiratory disease and presence of mental illness, because they frequency distribution was less than 10% of the total. Additionally, analysis of a single respondent in a household, rather than paired data, does not fully capture the entire picture of the family situation. Finally, Correlations between independent variables were not examined in detail. Therefore, we have not been able to confirm whether the effects are direct or indirect.

### Conclusions

This study family variables, such as family composition were found to be associated with family-related social problems during the COVID-19 pandemic. First, parents of preschool children were more likely to be anxious about the possibility of them abusing of their children. Furthermore, smokers were more likely to report perceiving a risk of being a victim of spousal abuse and domestic violence from spouse. Those whose income had also decreased following the COVID-19 pandemic, and those who were employed, and those with few rooms in their houses were more likely to report Internet dependency. Finally, mental health was impaired among those whose income was reduced following the COVID-19 pandemic and among members of families with conflicting opinions about COVID-19.

Although it is difficult to interpret these results as indicating direct causality, the results could help inform risk assessments for family support. For example, the presence of a preschooler or a smoker in the family is an indicator of the risk of violence in the family. Alternatively, assessment of reduced income and differences in attitudes toward COVID-19 in the family may provide options for intervening in cases of mental illness and family problems.

## Supporting information

S1 Data Anonymized data set. (XLSX)
